# Adult and Pediatric Nail Unit Melanoma: Epidemiology, Diagnosis, and Treatment

**DOI:** 10.3390/cells12060964

**Published:** 2023-03-22

**Authors:** Jade Conway, Jane S. Bellet, Adam I. Rubin, Shari R. Lipner

**Affiliations:** 1 School of Medicine, New York Medical College, Valhalla, NY 10595, USA; 2 Department of Dermatology and Pediatrics, Duke University School of Medicine, Durham, NC 27710, USA; 3 Department of Dermatology, Perelman School of Medicine at the University of Pennsylvania, Philadelphia, PA 19104, USA; 4 Hospital of the University of Pennsylvania, Philadelphia, PA 19104, USA; 5 Children’s Hospital of Philadelphia, Philadelphia, PA 19104, USA; 6 Department of Dermatology, Weill Cornell Medicine, New York, NY 10021, USA

**Keywords:** nail, nail unit melanoma, subungual melanoma, acral melanoma, longitudinal melanonychia, pediatric melanoma

## Abstract

Nail unit melanoma (NUM) is an uncommon form of melanoma and is often diagnosed at later stages. Approximately two-thirds of NUMs are present clinically as longitudinal melanonychia, but longitudinal melanonychia has a broad differential diagnosis. Clinical examination and dermoscopy are valuable for identifying nail findings concerning malignancy, but a biopsy with histopathology is necessary to confirm a diagnosis of NUM. Surgical treatment options for NUM include en bloc excision, digit amputation, and Mohs micrographic surgery. Newer treatments for advanced NUM include targeted and immune systemic therapies. NUM in pediatric patients is extremely rare and diagnosis is challenging since both qualitative and quantitative parameters have only been studied in adults. There is currently no consensus on management in children; for less concerning melanonychia, some physicians recommend close follow-up. However, some dermatologists argue that the “wait and see” approach can cause delayed diagnosis. This article serves to enhance the familiarity of NUM by highlighting its etiology, clinical presentations, diagnosis, and treatment options in both adults and children.

## 1. Etiology

Nail unit melanoma (NUM) is categorized as subungual, ungual, or periungual. Until recently, NUM was considered a variant of acral lentiginous melanoma (ALM), which accounts for 2–3% of all cutaneous melanomas [1,2,3].

NUMs are thought to originate in the nail matrix, the primary location of melanocytes in the nail unit, where the proliferation of atypical melanocytes leads to tumor growth [4]. A study analyzing four longitudinal nail biopsies taken from a normal fingernail and the nail of an accessory digit of two male white cadavers showed that the nail matrix, not the nail bed, is the primary location of nail unit melanocytes; all four melanocyte-specific stains (Tyrosinase, MITF, Melan-A, Sox-10) identified melanocytes within the proximal nail matrix while showing a complete absence of staining in the nail bed [4]. Infrequently, NUM originates in the epithelium of the nail bed or in the epidermis of the surrounding nail [5,6].

Our understanding of the etiology of ALM, and thus NUM, continues to evolve. Acute or chronic trauma has been proposed to play a role in the pathogenesis of NUM; most cases of NUM are localized to either the thumb or hallux and these digits are most frequently exposed to injury and trauma [7,8]. In a retrospective study of 406 patients with NUM, 58% of all cases of the fingers were localized to the thumb, while 86% of all cases on the toes were localized to the hallux [8]. In a comparative study of 87 patients with NUM, 21.8% recalled previous trauma history, and trauma-related cases were more likely to involve the toenail (*p* = 0.4) [9]. The role of trauma remains unclear because it is subject to recall bias and patients with trauma may visit physicians more frequently and thus be diagnosed [7]. Additionally, the thumb and hallux being larger digits with proportionally greater nail matrix surface area may also explain their more frequent localization. Unlike cutaneous malignant melanoma, NUM does not appear to be related to sun exposure. In an experimental study using a Dermalite UV machine as a source of radiation to measure nail plate penetration of 10 cadaver fingernails, the mean penetration of UV-A light was 1.65% while UV-B light was completely blocked by all fingernail plates [10]. The thick compact keratin layer of the nail unit is thus thought to act as a complete shield from sunlight or UV light [10], and many NUMs are reported in sun-shielded areas such as the toes of individuals who do not walk barefoot [7].

Mutations in BRAF, NRAS, and KIT and amplifications of CCND1, CDK4, MITF, and TERT were well established in cases of acral melanoma [11]. NUM was previously considered a subset of ALM, but it is now recognized that there are molecular and genetic differences between the two. A study analyzing the clinical and molecular features of 54 cases of NUM and 78 cases of ALM found that KIT mutations were more frequently found in NUM compared to ALM (16% vs. 3%), while KRAS mutations were predominantly found in NUM (5% vs. 0%). BRAF mutations occurred almost exclusively in ALM (22%) compared to NUM (3%), while NRAS mutations occurred in 37% of ALM compared to only 9% of NUMs [11]. 

In a study investigating the genomic profile of 122 acral melanomas using DNA sequencing, mutations in BRAF (21.3%), NRAS (27.9%), and KIT (11.5%) were identified. KIT mutations were present in 3/6 (50%) NUM cases, a significantly higher frequency when compared to non-NUM acral melanomas (*p* = 0.03); however, there was no association with BRAF or NRAS mutations [12]. In a retrospective cohort study investigating the genomic profiles of 29 acral, mucosal, and vulvovaginal melanomas using whole transcriptome mRNA/DNA sequencing, as well as mRNA expression profiling and UV signature analysis, alterations in BRAF were detected in 36%, compared to the mucosal and vulvovaginal melanomas showing no alterations (*p* = 0.0159); the study did not separate NUM from other non-NUM acral melanomas [13]. Additionally, acral melanomas showed a higher frequency of UV-induced mutations and overexpression of ERBB2 aberrations (*p* < 0.01), although it was not specified which were nail and non-nail melanomas.

## 2. Risk Factors

Although ALM prevalence is similar between different racial and ethnic groups, it represents 1–2.5% of all melanomas in white individuals, compared to 15–35% in dark-skinned and 50–58% in Asian individuals [3,7,14]. The literature is lacking regarding racial differences for NUM specifically, NUMs are most frequently diagnosed between the ages of 50 and 70 years, though extremely rare cases have been reported in children [7]. 

Genetic risk factors may also contribute to NUM risk. In a large cohort study examining the cumulative risk of melanoma in 238,724 first-degree relatives of 46,091 melanoma patients, there was an increased risk of any melanoma subtype in patients with first-degree relatives diagnosed with ALM [2,8], suggesting that ALM shares genetic factors with other melanoma subtypes. In a retrospective study of 978 patients with cutaneous melanoma, individuals with ALM had secondary cancers or a family history of cancer more often compared to patients with other melanoma subtypes. Although the etiology of NUM remains unclear, it is most likely multifactorial. 

## 3. Clinical Presentation & Dermoscopy

**Figure 1:**
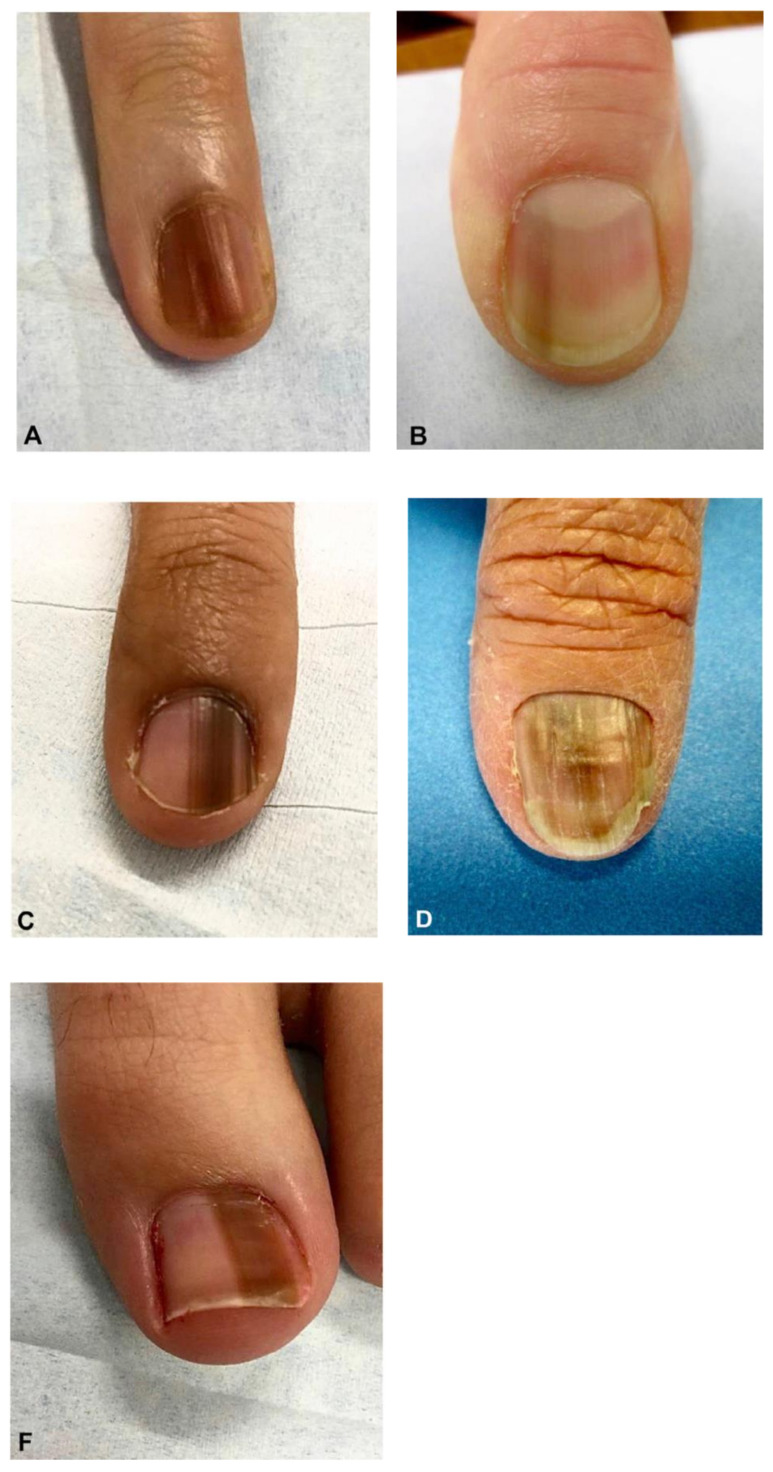
Clinical appearance of subungual melanoma cases. (**A**), The patient is a 26-year-old female with a 5-mm brown band (68% width percentage) on the nail of the right ring finger. (**B**), The patient is a 63-year-old woman with a 2.5-mm brown band (18.6%) on the right thumbnail. (**C**), A 42-year-old man with a 6-mm brown band (54.5%) on the nail of the left fifth finger. (**D**), A 52-year-old woman with a 7.5-mm brown band (60.0%) on the left thumbnail. (**F**), An 18-year-old woman with a 5.5 mm dark brown band (46.4%) on the nail of the left hallux. Reproduced with permission from ref. [19].

**Figure 2:**
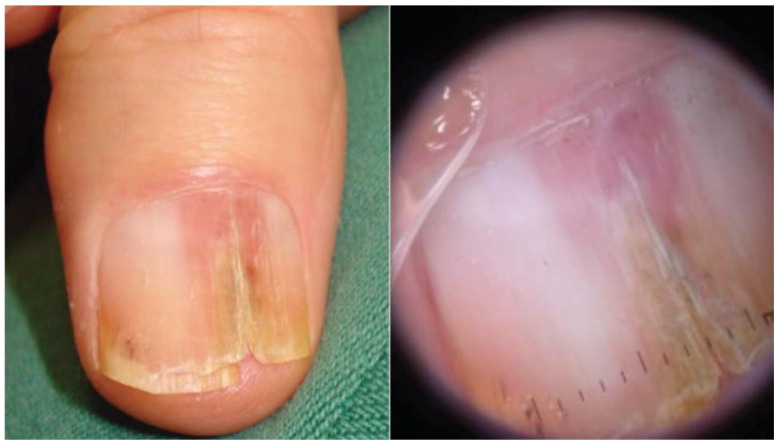
Invasive melanoma in a band of LLE. Dermoscopy highlights splinter hemorrhages and distal triangular onycholysis. Reproduced with permission from ref. [20].

**Figure 3:**
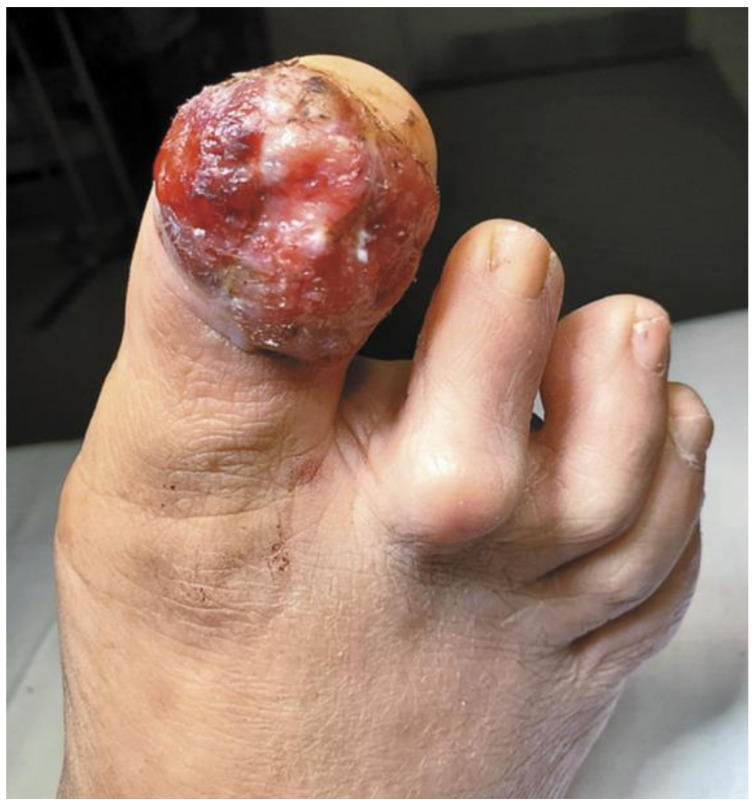
A Rare Case of Osteoinvasive Amelanotic Melanoma of the Nail Unit; clinical photo demonstrating a hypervascular, nonpigmented lesion on the dorsum right distal hallux. Reproduced with permission from ref. [21].

Approximately two-thirds of NUMs are present clinically as longitudinal melanonychia (LM), defined as a longitudinally oriented band of brown to black pigment extending the length of the nail plate [15,16] (Figure 1). However, up to 25% of nail melanomas are amelanotic, presenting with a pink, red, or flesh-colored papule, or longitudinal erythronychia often accompanied by onycholysis, notching, splitting, bleeding, or ulceration [17,18] (Figure 2 and Figure 3). NUM localizes most frequently in the nails of the thumb, hallux, and index fingers, but has been reported in all digits [16]. 

For LM presentation, the ABCDEF rule of NUMs, is much more complicated than the mnemonic used for cutaneous melanomas and has been proposed to aid in clinical evaluation. *A* stands for Age (peak age is 50–70 years), as well as African Americans, Asians, and Native Americans (in whom NUM can account for up to 50% of all melanomas) [22]. *B* stands for Bands with breadth > 3 mm, Brown-black discoloration, and irregular Borders. *C* stands for Change while *D* stands for the Digit most commonly involved (thumb > hallux > index finger) and *E* stands for Extension of pigment. Finally, *F* stands for Family history of melanoma or dysplastic nevi [22]. 

On physical examination, clinical findings include band width measuring greater than 3 mm, or more than 40% of the total nail plate width. In a retrospective cohort study evaluating the clinical features of 84 patients who underwent nail biopsy for LM, the mean band width for benign LM was 3.11 mm compared to 5.31 mm for NUM cases (*p* = 0.002); mean width percentage of the nail was 24.84% for benign LM compared to 49.48% for NUM cases (*p* < 0.001) [19]. When comparing complete sets of data on ABCDEF criteria for 27 patients in this study (20 benign, 7 NUM), the average number of criteria met for benign LM cases was 2.45 compared to 2.86 for NUM cases, showing no significant difference between the two groups. The most common positive criteria for the NUM group were “B” (87.5%) and “C” (71.4%) [19]. Based on this data, the ABCDEF criteria, which has never been previously validated, may not be a useful or reliable mnemonic for NUM. 

**Figure 4:**
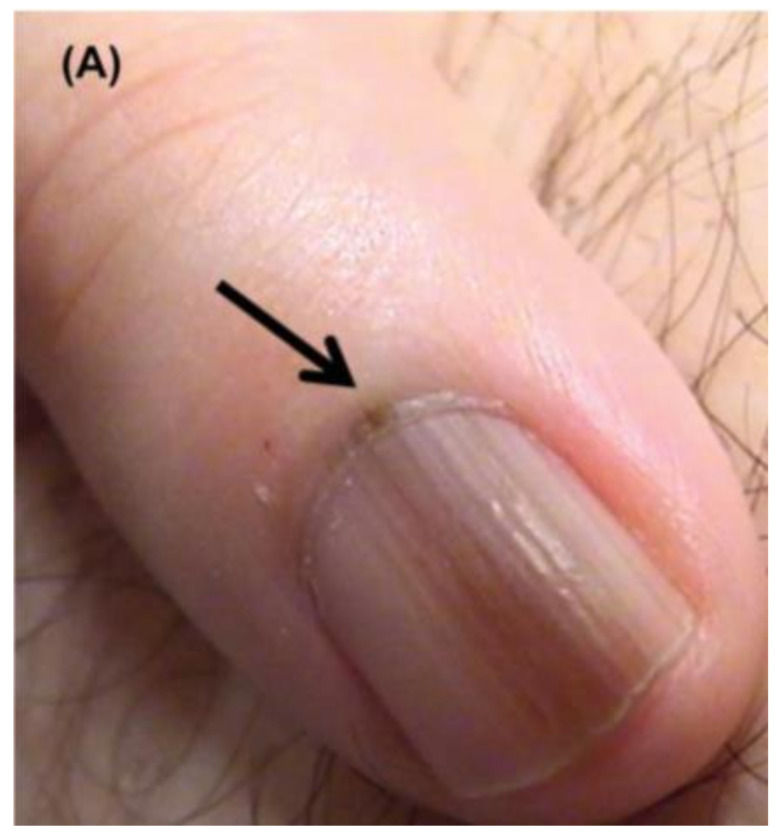
Nail unit melanoma clinical features—LM and Hutchinson sign (arrows). Reproduced with permission from ref. [3].

Additional concerning features include color band heterogeneity, band darkening or widening, a triangular band (wider proximally compared to distally), purulence or bleeding, nail splitting, and a Hutchinson’s sign, defined as an extension of pigment onto the adjacent skin [7,17] (Figure 4). Pigment on the surrounding skin is not pathognomonic for malignancy and must be distinguished from similar non-pathologic pigmentation caused by congenital melanocytic nevi, Laugier–Hunziger syndrome, or drug-induced exogenous pigmentation [17,23]. Color intensity does not accurately indicate whether an LM is benign or malignant as both NUM and benign LM can vary from dark black to very light brown [7]. 

Although NUM is part of the differential diagnosis for LM, there are many benign etiologies, including fungal melanonychia and bacterial infection, such as pseudomonas aeruginosa, exogenous pigment, subungual hematoma, non-melanocytic tumors, and drugs, as well as melanocytic etiologies, including benign melanocytic activation, lentigo, and nail unit nevi [24]. Melanocytic activation is the most common cause of LM in adults [16], but it can sometimes be difficult to distinguish LM secondary to a benign process from LM due to melanoma based on physical examination alone. Whether single vs. multiple digits are affected can impact physician concern, as it is common for darker-skinned individuals to exhibit LM on multiple nails; almost 100% of individuals with skin types V–VI will demonstrate this finding by the age of 50 [16].

**Figure 5:**
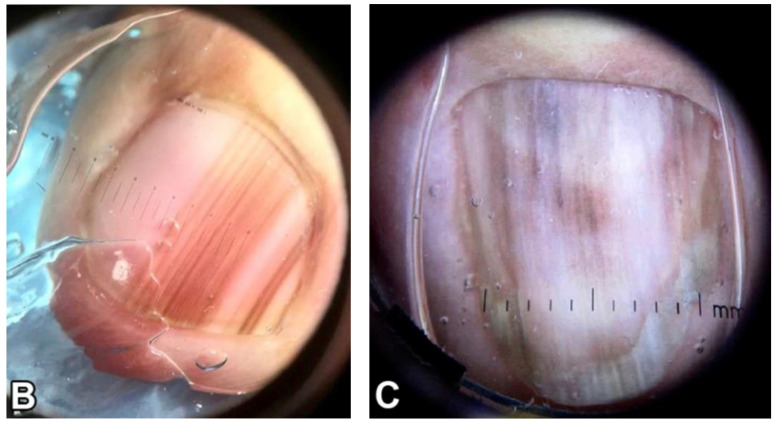
Dermoscopic appearance of subungual melanomas. (**B**), Brown lines on a brown background, irregular color, thickness, and spacing with no loss of parallelism. (**C**), Brown lines on a brown background, irregular color, thickness, and spacing with loss of parallelism. Reproduced with permission from ref. [19].

**Figure 6:**
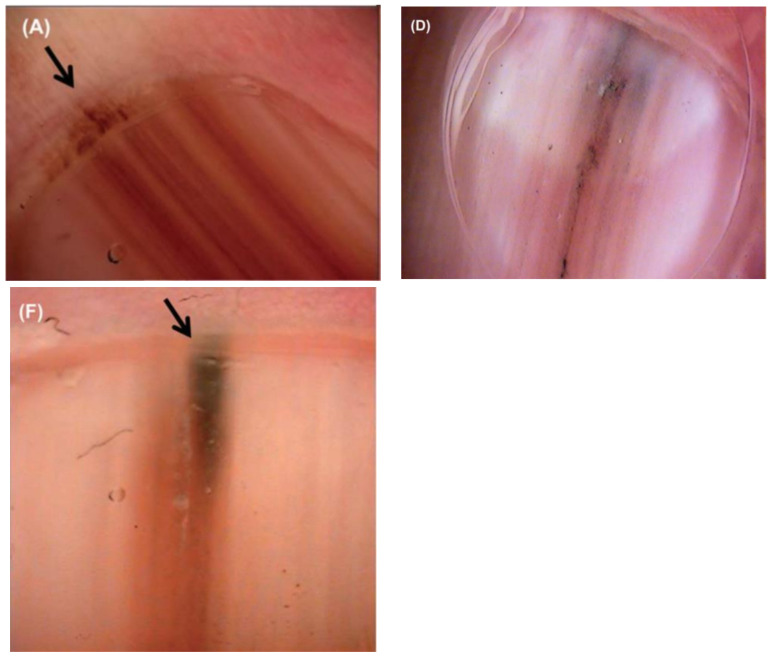
(**A**,**D**,**F**), Nail unit melanoma dermoscopic features—brown bands irregular in color, width, and spacing, and Hutchinson sign (arrows). Reproduced with permission from ref. [3].

Classic dermoscopic findings for NUM presenting as LM are a brown to black background associated with longitudinal lines that are irregular in color, width, and spacing, with a loss of parallelism [3,17,19,25,26] (Figure 5 and Figure 6). In a cohort study of 19 patients with biopsy-proven NUM in situ, asymmetry (OR = 34), presence of Hutchinson’s sign (OR = 18.18), multicolor (OR = 11.59), border fading (OR = 9.33), and width of at least 3 mm (OR = 5.31) were significantly associated with NUM in situ when compared to 26 cases of benign melanonychia [27]. In a study evaluating the dermoscopic features of 23 cases of biopsy-confirmed NUM, 60% of cases presented LM, 60.8% showed irregular nail plate changes, 52.1% showed blurred borders, and 43.4% had irregular blood vessels. Cases of amelanotic NUM showed eroded nodules or granulated masses of the nail bed, often accompanied by whitish to milky red veils or peripheral yellow-to-brown scaling and crusts; vascular polymorphisms with irregular vessels were also common [24]. Only 80% of the cases presenting as LM showed features suggestive of malignancy, while the remaining 20% had clinically benign features [24]. Therefore, although dermoscopy is a helpful tool for recognizing clinical features suggestive of malignancy, it is not sensitive enough to detect all cases of NUM. For this reason, matrix biopsy with histopathologic examination remains the gold standard for diagnosis [3,17]. 

It is important to consider that clinical and dermoscopic findings, particularly for LM, can differ based on skin type and that melanocytic activation occurs more frequently in patients with darker skin tones [28]. In a 10-year retrospective cohort study of 248 cases of benign LM, darker-skinned patients had higher band width percentage (*p* = 0.0125), lower band brightness (*p* < 0.001), and more band changes (*p* = 0.0071) compared to lighter-skinned patients. Darker-skinned patients vs. lighter-skinned patients also had more brown vs. gray coloration on dermoscopy (*p* = 0.0232) [28]. 

## 4. Diagnosis

History, clinical examination, and dermoscopy are necessary, but not always sufficient for diagnosing NUM. Diagnosis of NUM is often delayed. In a retrospective cohort study of 84 patients (8 cases of NUM, 76 benign cases) who underwent nail biopsies for LM, patients with NUM had their bands for a longer period (mean duration 128.9 months) compared to patients with benign melanonychia (mean duration 38.3 months) (*p* = 0.017) [19].

Reasons for the delay include lack of a structured clinical approach to pigmented nail lesions, varied presentation with a high incidence of amelanotic melanoma, absence of nail changes during the radial growth phase, inadequately performed biopsies, and histopathologic misinterpretation [29,30,31]. Both physician and patient education play important roles in the timely identification of nail changes; however, physicians may lack experience in diagnosing nail conditions and patients may not be knowledgeable about nail signs and symptoms that should prompt an office visit. In a nationwide study surveying 402 attending and resident dermatologists on the management of LM, 54.2% of respondents stated they were “fairly” confident in assessing LM while 28% stated they were “not confident” and only 17.8% of respondents stated they were “very confident” (*p* < 0.0001) [32]. Only 18.2% of dermatologists surveyed reported performing nail examinations at each visit, and 58% performed them only as a component of a total body skin examination [32]. In a study surveying 363 patients in outpatient clinics, only 5% of patients had heard of the ABCDEF mnemonic for NUM in contrast to 9.9% having heard of the ABCD mnemonic for cutaneous melanoma; only 1.4% of patients reported being counseled about the ABCDEF mnemonic for NUM by their physicians compared to 13.8% of patients that were advised about the ABCD mnemonic for cutaneous melanoma [33]. Additionally, only 31.4% of patients stated they assessed their nails for color changes; 10.2% of patients reported having LM, with only 45.9% of those patients seeking medical attention [33]. In a study designed to assess and score the accuracy of 27 internet sources relating to NUM, websites varied in accountability and quality while readability was poor overall (mean overall score 16.1/40, range 7–25) [34]. Diagnosis of LM requires in-person examination and is not amenable to telemedicine visits, which could also account for the delay in diagnosis. Evaluation of LM requires physical examination with precise measurement of the band width and width of the entire nail plate and dermoscopy, and thus requires an in-person visit [35,36].

Physician uncertainty may also cause delays in diagnosis, with a broad range of differential diagnoses for LM. These include benign causes, such as melanocytic activation (secondary to onychotillomania, onychophagia, friction, and medications), lentigo, nevus, hematoma, onychomycosis, and bacterial infections [3]. Exogenous pigment on the nail surface can also be mistaken for NUM, with a variety of causes such as dirt, discoloration due to smoking, potassium permanganate, tar, and silver nitrate [7]. Amelanotic NUM may mimic lichen planus, subungual fibrokeratoma, keratoacanthoma, squamous cell carcinoma, pyogenic or foreign body granuloma, glomus tumor, onychopapilloma, or verruca vulgaris. If amelanotic NUM manifests as an eroded nodule with purulence, it may be incorrectly dismissed as an ingrown nail or infected wart [7]. 

For LM cases, a nail matrix biopsy is the ideal way to distinguish between benign and malignant causes of melanonychia. However, nail clipping can be a useful, non-invasive tool to aid in surgical planning, as it can help localize pigment within the nail plate and can be used as a rapid triage maneuver to detect cases in which urgent nail matrix biopsy is needed [37]. A pigment found in the dorsal aspect of the nail plate localizes lesions to the proximal matrix, while pigment in the ventral aspect of the nail plate localizes to the distal matrix [38]. Proper localization in cases of uncertainty may help to avoid permanent post-procedure nail dystrophy. In addition to aiding in surgical planning, nail clipping can rule in or rule out other differential diagnoses, such as fungal melanonychia, pseudomonas, and hematoma. In a case of a 64-year-old female with a history of melanoma, histological sections of a nail clipping of the left great toenail performed to confirm onychomycosis revealed the presence of intervening round spaces with melanin pigment, some of which included enlarged nuclei; due to this finding, additional specimens were obtained which ultimately made the diagnosis of NUM in situ [30]. Physicians should perform further clinicopathologic testing when melanocytes or melanocyte remnants are identified within a nail clipping. 

The biopsy technique is decided based on the location of the band (i.e., lateral, medial) and whether the pigment is in the proximal or distal nail matrix [39,40]. In order to comprehensively perform a biopsy on the nail unit for LM, sampling of the nail matrix is required; this site is normally protected by both the nail plate and the overlying proximal nail fold [41]. As the proximal matrix is responsible for the majority of nail plate production, it is more susceptible to scarring or split development of the nail following nail matrix biopsy [41]. When the mid-portion of the nail plate is involved, there is a high risk for postoperative nail dystrophy. In particular, pigment localized to the dorsal nail plate will require a biopsy including the proximal nail matrix which increases the risk of scarring [39,40].

For cases of LM less than 3 mm wide originating in the distal matrix, a full thickness 3 mm punch biopsy of the nail matrix is acceptable, but the shave matrix technique is still preferred by most nail specialists [41]. For low-risk toenail LM, a punch biopsy may be reasonable and result in less onychodystrophy. Cases of LM greater than 3 mm in width in the mid-nail plate or originating in the proximal matrix are more challenging, and require a tangential shave technique in which a very thin tissue sample is taken from the matrix epithelium and a significant portion of the dermis [39,40]. A lateral longitudinal excisional biopsy provides adequate sampling in cases of wider, laterally located LM [39,40]. This is a more invasive and technically demanding procedure, as all incisions are carried down to the level of the bone; adequately performed procedures will remove the entire lateral matrix horn but can occasionally leave small remnants behind that can cause post-operative cysts, spicules, and pain [41]. Nail malalignment is a potential side effect patients should be counseled on with a lateral longitudinal excision.

Cases with LM broader than 6 mm or diffuse melanonychia may require multiple punch biopsies, a transverse matrix incisional biopsy, or en bloc excision as preliminary investigation [41]. If the proximal nail fold (cuticle) has pigmentation (Hutchinson sign), a shave or punch biopsy may be performed and submitted with the specimen [41]. 

## 5. Histopathology

It can be both clinically and histopathologically challenging to distinguish benign and malignant LM. Immunohistochemical staining has been suggested to aid in identifying NUM. In a retrospective study comparing 14 cases of benign subungual melanocytic proliferation with 13 cases of NUM in situ, 92.9% of patients with benign melanonychia showed completely negative staining for Preferentially Expressed Antigen in Melanoma (PRAME) while 76.9% of patients with NUM in situ exhibited >50% immunostaining for PRAME [42]. In another study evaluating whether PRAME immunoreactivity can differentiate between benign and malignant nail lesions, PRAME expression was significantly higher in cases of NUM (*p* < 0.0001); of 18 NUM cases, 61.1% showed >75% PRAME-positive melanocytes [43]. These results suggest PRAME can be a tool for clinicopathologic diagnosis of NUM. On account of the occasional discordance of PRAME status and ultimate diagnosis reported, it is important to consider PRAME status as just one factor which contributes to the final diagnosis. Clinical integration and integration with the histopathologic features in the specimen are essential when considering PRAME status in the final histopathologic diagnosis.

In addition to PRAME, there are other immunohistochemical stains commonly used for cutaneous melanomas that can be helpful in the diagnosis of NUM. In a retrospective study examining the histological and immunohistochemical features of 65 cases of NUM in situ, Melan-A (also known as MART-1), human melanoma B (HMB) 45, and mouse monoclonal melanoma antibody (PNL2) showed superior diagnostic sensitivity compared to S-100 protein (>95% vs. 83.1%) [44]. According to an international survey of the European Nail Society and Council for Nail Disorders, Melan-A/MART-1 continues to be the preferred method for the identification of melanocytes in the nail unit [45]. Pathological interpretation can be difficult, as the nail unit’s tissue is fragile, specimens may be small or superficial, and tissue is at risk of suboptimal grossing. It is imperative that physicians collect adequate samples to avoid only sampling peripheral areas where dermal-invasive tissue may not be apparent, leading to underestimation of disease extent [46]. 

**Figure 7:**
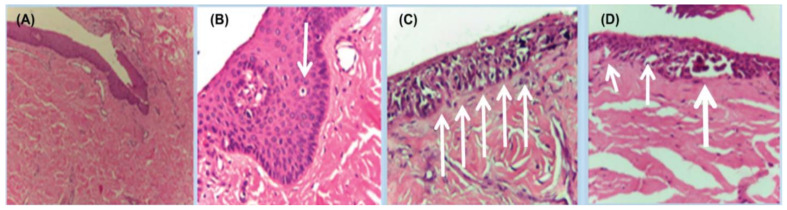
Histopathology of in situ matrix melanomas. Hematoxylin and eosin stain. (**A**), Almost invisible changes. (**B**), A few cells are obvious (arrow). (**C**), The entire matrix epithelium shows pagetoid spread of atypical melanocytes (arrows). (**D**), Lentiginous (small arrows) and nest-like (large arrow) proliferation of atypical melanocytes. Reproduced with permission from ref. [3].

**Figure 8:**
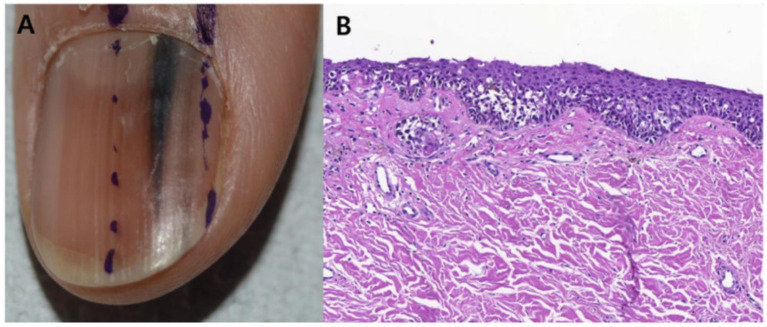
Representative clinical morphology, photomicrographs of H&E staining, and IHC staining for cyclin D1 and PRAME of selected subungual melanoma in situ. One 44-year-old patient (**A**), 2 mm-wide melanonychia showed (**B**), atypical melanocyte proliferation with confluency and pagetoid spread (200× magnification, H&E). Reproduced with permission from ref. [42].

Histopathologic clues for the diagnosis of NUM include lentiginous growth of single melanocytes predominating over nests, increased numbers of single junctional melanocytes, prominent pagetoid spread, increased cellular atypia, mitotic activity, and junctional lymphocytic inflammatory response [47,48,49,50,51] (Figure 7 and Figure 8). In Park et al.’s study analyzing the histopathologic features of 18 cases of NUM in situ from 2005–2014 to assess for diagnostic clues, 100% of cases showed an uneven distribution of solitary melanocytes with irregularly scattered atypical melanocytes and 67% of cases (12/18) showed focal pagetoid spread [48]. Cases varied in melanocyte density, defined as the number of melanocytes over a 1 mm epithelial-dermal junction; 78% (14/18) measured more than 40, but 3 cases showed less than 30 [48]. In Izumi et al.’s study analyzing the histopathologic findings of 50 cases of NUM, 52.6% (26/50) of cases showed the proliferation of atypical melanocytes in scattered solitary units [49]. 

Tan et al. conducted a similar study looking at the histopathologic features of 124 cases of NUM from 1951–2004, in which 28 cases were NUM in situ. Compared to acral nevi, NUM in situ lesions showed a predominance of solitary melanocytes over nests (96% vs. 64%, *p* = 0.0001), moderate-to-marked cellular atypia (96% vs. 0%, *p* < 0.0001), and a moderate-to-marked lymphocytic infiltrate (43% vs. 4%, *p* < 0.0001) [50]. 

Amin et al. studied the histopathologic features of 20 nail unit melanomas and 15 benign subungual melanotic lentigines to compare histopathologic parameters for diagnostic distinction. Compared to benign melanonychia, invasive and non-invasive NUMs were significant for high melanocyte count (M = 102 and M = 58.9 vs. M = 15.3), confluence of melanocytes (0% vs. 100%), multinucleation of melanocytes (0% vs. 85%), inflammation at the epithelial–stromal interface (0% vs. 45%), and moderate-to-severe atypia of melanocytes (5% vs. 90%) (*p* < 0.001) [51]. 

The American Joint Committee on Cancer (AJCC) uses the TNM staging system for all cutaneous melanomas, without a separate staging system for NUM. Breslow thickness and ulceration status are used to determine the extent of tumor invasion, which indicate disease severity. Clark level was previously included as another measure of tumor invasion but is no longer used by the current AJCC system [52]. Breslow thickness is a quantitative measure of depth from the granular layer of the skin down to the deepest point of a tumor, while the Clark level was a more descriptive term that characterizes the anatomical layer of skin involved by the tumor (Level I epidermis, level II papillary dermis, level III papillary-reticular dermal interface, level IV reticular dermis, level V subcutaneous tissue). As the nail matrix typically lacks a granular layer, precise measurements of Breslow thickness are more difficult compared to measurements in the skin [31]. In a case series of 124 diagnosed NUMs, the median Breslow thickness was 3.2 mm (range: 0.25–14 mm) while 79% of tumors were locally advanced at presentation with Clark level IV or V [50]. In another cohort of 103 patients diagnosed with in situ or invasive NUMs of the hand, the median Breslow thickness of invasive tumors (n = 94) was 3.1 mm and 43% of patients presented with Clark level IV invasion [53]. In a study analyzing the tumor-to-bone distance in 30 cases of invasive NUM, bone attachment or invasion was more likely when tumor thickness measured greater than 4 mm (*p* = 0.033) [54]. 

## 6. Management

### 6.1. Management: Surgery

Treatment of NUM is challenging because of the unique anatomy of the nail apparatus, the tendency for rapid horizontal growth, the short matrix-to-bone distance, and the frequent irregular borders of NUM [29,46,54]. Excision of the nail matrix is challenging, with the risk of damaging the subjacent extensor tendon or leaving behind matrix epithelium that can lead to local recurrence or spicule formation [45]. It is essential that physicians be familiar with these risks during surgical planning in order to avoid complications. 

Historically, radical amputation at the level of the metacarpal/metatarsal bones was advocated as the best treatment option for all NUMs based on efforts to reduce the risk of recurrence or metastases [29,55,56]. However, this approach has more recently become increasingly controversial as most previous studies or cases examining surgical treatment outcomes do not account for individual factors at presentation or tumor depths (melanoma in situ vs. invasive) and thus have not accurately reported outcomes [55]. Recent studies have proposed more conservative options for surgical management that prioritize the complete removal of tumors as well as the quality of life and digital function of patients [55,56].

**Figure 9:**
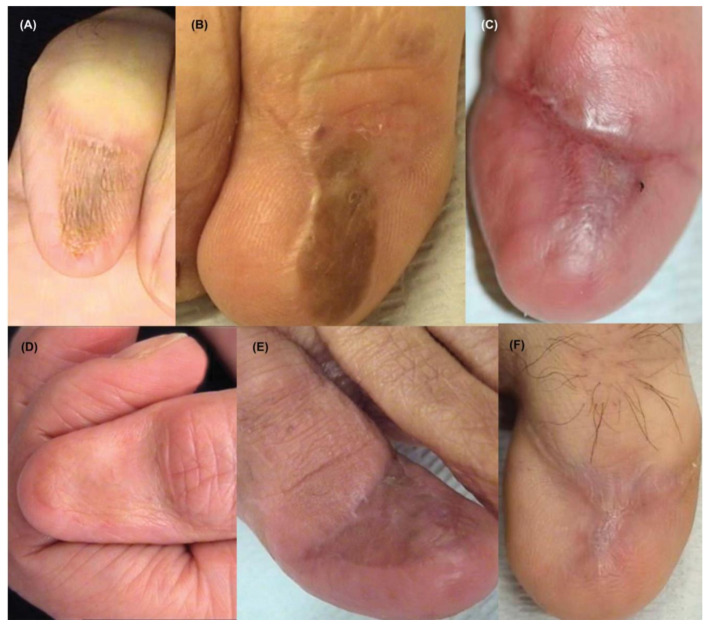
Nail unit melanoma after surgical outcome—good aesthetic and functional outcome. (**A**,**B**,**D**,**E**) defects required full-thickness skin graft for closure. (**C**,**F**), were allowed to heal secondarily. Reproduced with permission from ref. [3].

For surgical excision of in situ NUM, the entire nail unit including the nail plate, nail bed, and nail matrix must be removed. A split or full-thickness skin graft may be used to repair the resulting defect or may heal by second intention [39] (Figure 9). Most treatment and surgical guidelines for NUM were adapted from those of cutaneous melanoma. Though guidelines on radial/peripheral margins exist, there is limited data on deep margins; peripheral margins are either narrow (1–2 cm) or wide (3–5 cm) [31]. In a retrospective study analyzing 7 cases of in situ or minimally invasive NUM treated with conservative surgical excision, all cases showed no recurrence after a mean follow-up of 45 months (minimum 24 months) [57]. All patients were managed with 5–10 mm safety margins followed by full-thickness skin grafting and reported good cosmetic and functional outcomes. Given these results, as well as 62 other cases found in the literature, the investigators recommended non-amputative conservative excision as a treatment for in situ NUM [57]. A meta-analysis of 5 studies with 109 patients treated for in situ or minimally invasive NUM reported no difference in local recurrence rates when comparing digit amputation to conservative functional surgery treatment (OR = 1.57, 95% CI: 0.31–8.00, statistical heterogeneity I^2^ = 0%) [58]. In a retrospective comparative analysis of 62 patients with stage I or II NUM, patients received either amputation proximal to the distal interphalangeal joint or local excision of tumors with or without bone resection; recurrence occurred in 48.4% of amputation patients compared to 35.5% of functional surgery patients [56]. Five-year survival rate was 66.5% for patients who underwent amputation and 91.7% for those who underwent functional surgery. Conservative excision of non-invasive NUM did not adversely affect prognosis when compared to amputation and is thus recommended in order to avoid unnecessary post-operative functional deficits [56]. 

For invasive NUMs, the thickness and location of the tumor dictate the surgical approach. Complete surgical removal of the entire tumor is the priority, but physicians must also take into account functional preservation. For example, lesions located on the hallux or thumb require special consideration as these digits carry higher functional importance in terms of preserving balance and grasping abilities for patients [39]. Amputation of the thumb at the level of the carpometacarpal joint, metacarpal bone, metacarpophalangeal joint, and interphalangeal joint disable the hand by 38%, 37%, 36%, and 18%, respectively [17,59,60].

Advanced cases of NUM involving the bone or joint spaces may not be suitable for treatment with functional surgery and warrant amputation. In a retrospective study of 116 patients with NUM who underwent amputation, univariate analysis showed that the level of resection was not significantly associated with overall or disease-specific survival on the thumb (*p* = 0.67, *p* = 0.68), fingers (*p* = 0.39, *p* = 0.16), hallux (*p* = 0.36, *p* = 0.56), or toes (*p* = 0.32, *p* = 0.22) [61]. If surgeons choose to perform amputations, they may consider a more conservative approach with amputation at the most distal joint possible in order to avoid unnecessary disability in patients. 

As peripheral margins of NUM are often difficult to delineate, the ability to confirm tumor-free margins histologically during surgery is appealing. Several studies support the use of Mohs Micrographic Surgery (MMS) as a digit-sparing approach for the treatment of NUM, particularly for tumors with a Breslow depth of less than 2 mm [17]. In a retrospective analysis of 14 patients with NUM (both in situ and invasive) treated with MMS, histologically tumor-free margins were obtained in all patients; the standard frozen tissue sectioning technique was used in all cases, with some of the more recent cases employing HMB-45 immunohistochemistry. Eight (57%) tumors were cleared in a single stage, while four (29%) required two stages and two (14%) required three or four stages of excision [62]. Only three (21.4%) patients experienced marginal recurrence and required re-excision, and this did not affect overall patient outcome. The average depth of all cases (4 in situ, 10 invasive) was 0.98 mm (range: 0–3.3 mm), and all cases were treated with a minimum of 6 mm peripheral margins [62]. 

Another retrospective series analyzed 62 cases of NUM treated with MMS with frozen section hematoxylin and eosin alone, hematoxylin and eosin with HMB-45 immunohistochemical stains, or MART-1 immunohistochemical stains; only five (8.2%) cases had local recurrence and no cases that utilized MART-1 staining during MMS had local recurrence [63]. Local recurrence-free survival rates at 5 and 10 years were 91.8% and 82.6%, respectively and 96.5% of patients avoided amputation [63]. In a more recent retrospective observational study of 14 patients with in situ NUM treated with MMS with MART-1 immunostaining, only 1 (7.1%) patient developed recurrence at 6.6 years and was subsequently treated with amputation; nine cases (64.3%) were cleared in a single stage while four (28.6%) required two stages and one patient requires four stages [64]. Accurate histopathologic evaluation of the nail unit tissue is essential for the successful treatment of NUM with MMS. MART-1/Melan-A immunostaining of the stages is preferred for the interpretation of melanonychia, and long-term post-procedure follow-up is necessary to identify recurrences [45]. 

### 6.2. Management: Adjuvant Therapy

#### 6.2.1. Targeted Therapies

In addition to surgical removal of tumors, patients with advanced cases of NUM may be candidates for newer targeted and immune therapies (Table 1 and Table 2). BRAF mutations are more common in non-acral melanomas (50% vs. 20%) and occur in only 15–20% of acral melanomas. BRAF (vemurafenib, dabrafenib, encorafenib) and MEK (trametinib, cobimetinib) inhibitors have been well studied for the treatment of cutaneous melanomas, with limited data for the treatment of acral melanoma (NUM specifically) [65]. However, because of the positive clinical response of BRAF-positive metastatic cutaneous melanomas to BRAF and MEK inhibitor therapy, these treatments may be considered potential targets in NUM treatment. In a study of 13 patients with acral melanoma harboring BRAF V600E mutations treated with vemurafenib, the overall response rate (ORR) and disease control rate (DCR) were 61.5% and 92.3%, respectively; median progression-free survival (PFS) and overall survival (OS) were 5.4 (95% CI: 3.5–8.7) and 11.7 (95% CI: 8.1–23.6) months [65]. 

Several receptor tyrosine kinases are involved in melanoma growth and metastasis. Tyrosine kinase inhibitors imatinib mesylate and nilotinib may be useful for sustained remission; however long-term toxicity is not well studied. Sunitinib and dasatinib are not recommended for the treatment of NUM secondary to poor tolerability [65].

High-dose interferon alfa-2b (HDI) enhances cell-mediated cytotoxicity by directing lymphocytes to cancer cells and is FDA-approved for adjuvant therapy of resected high-risk melanoma. In a retrospective single-center study of 27 patients with stage III resected melanoma (20 acral, 7 cutaneous) receiving adjuvant HDI therapy, the 20 acral melanoma patients showed a 6-month recurrence-free survival (RFS) of 90% (95% CI, 76.9–103.1). Adverse events included fever, fatigue, and hepatotoxicity and resulted in 7.4% discontinuing treatment and 44.4% delaying treatment; 29.63% of patients received dose reductions [65]. Because of the lack of data on HDI for the treatment of NUM, better-studied agents are recommended until efficacy and safety for HDI are established.

#### 6.2.2. Immunotherapies

The development of immunotherapeutic agents to target key steps in the surveillance of tumors cells shows great promise for the treatment of NUM. Antibodies that target cytotoxic T-lymphocyte-associated antigen 4 (CTLA-4) such as ipilumumab and tremelimumab have been developed to enhance T-cell function and induce tumor regression [65]. Monoclonal IgG4 antibodies directed against programmed cell death protein-1 (PD-1), expressed at high levels in melanomas, block the PD-1-ligand interaction resulting in anti-tumor T-cell activation. Current PD-1 inhibitors include nivolumab and pembrolizumab [65].

Ipilumumab is FDA-approved as adjuvant therapy for patients with melanoma with regional lymph node metastases with previous complete resection. Nivolumab is approved for adjuvant treatment in patients who have undergone resection of melanoma and resection of all sites of disease. Pembrolizumab is approved for adjuvant therapy in melanoma patients with lymph node involvement who have undergone complete resection [66].

PD-1 inhibitor monotherapy, or in combination with lymphocyte activation gene-3 inhibitor (relatlimab) or CTLA-4 inhibitors is the standard of care for advanced cutaneous melanoma [67]. However, while immune checkpoint inhibitor therapy has become a first-line treatment option for patients with advanced cutaneous melanoma, standardized treatment guidelines for patients with NUM are lacking as most clinical trials do not report ALM or NUM outcomes separately [65,67,68].

Anti-PD-1 monotherapy may have better efficacy in advanced ALM patients compared to anti-CTLA-4 monotherapy, although both are well tolerated [65,69]. A systematic review of immune checkpoint inhibitors used in advanced ALM showed that the objective response rate of anti-CTLA-4 monotherapy was 11.4–25% (median OS > 7.16 months) compared to an objective response rate of 14–42% (median OS >14 months) for anti-PD-1 monotherapy; a single study investigating combination therapy showed an increased objective response rate of 42.9% [69]. Additionally, a meta-analysis of 19 studies with 646 patients with metastatic ALM exploring systemic treatment outcomes showed that patients treated with anti-PD-1 monotherapy had higher rates of OS (53%) compared with anti-CTLA-4 monotherapy (34%) (*p* < 0.001) [68]. PD-1 inhibitors should be considered first-line treatment for invasive NUM, while anti-CTLA-4 therapy can be considered in cases of intolerance or contraindications to anti-PD-1 therapy [65].

Patients with ALM respond differently to immune checkpoint inhibitors based on tumor location. In a retrospective study of 193 patients with advanced ALM (nail = 70, palm and sole = 123) treated with anti-PD-1 antibody, the objective response rate was significantly lower in the nail unit (8.6%) compared to in the palm or sole (21.1%) (*p* = 0.026); median overall survival was also significantly shorter in nail patients (12.8 vs. 22.3 months, *p* = 0.03) [70].

Despite advances in melanoma treatment, patients with ALM have limited response to current treatment options and subsequent worse overall survival rates compared to patients with other cutaneous melanomas [71]. Lower response to immune checkpoint inhibitors may be caused by decreased numbers of tumor infiltrating lymphocytes; lower PD-1 expression; and lower mutational burden of ALM compared to other melanoma subtypes [17]. Further research investigating therapies and response rates for NUM specifically is needed.

### 6.3. Management: Neoadjuvant Therapy

For patients with regional lymph node metastases, neoadjuvant treatment prior to undergoing resection may be appropriate. This approach may provide patients with the advantage of a stronger immune response and a reduction of tumor burden. In a phase II trial investigating neoadjuvant therapy in 86 melanoma patients (type unspecified), patients were split into 3 arms of treatment; Arm A received 2 × ipilimumab 3 mg/kg and nivolumab 1 mg/kg, arm B received 2 × ipilimumab 1 mg/kg and nivolumab 2 mg/kg, and arm C received 2 × ipilimumab 3 mg/kg followed by 2 × nivolumab 3 mg/kg [66]. Pathologic response rates, which were a strong marker for relapse-free survival, were 80% and 77% in arms A and B with complete response rates of 43% and 57%, respectively. Arm C was closed secondary to high toxicity. The researchers recommended that ipilimumab 1 mg/kg with nivolumab 3 mg/kg be further studied. Overall, the use of immune checkpoint inhibitors as neoadjuvant therapy shows promising results, but further studies, specifically for the treatment of NUM, are needed [66].

### 6.4. Prognosis by Location

There is limited data on the impact of NUM localization (fingernails vs. toenails) on overall prognosis. Since NUM of the toenails may not be as easily noticed by patients compared to fingernail NUM, it may be hypothesized that this would contribute to more profound diagnostic delays and overall survival rates for toenail vs. fingernail NUMs. However, in a retrospective single-center study analyzing data from 42 patients diagnosed with NUM, univariate analysis for overall survival showed prognosis was not significantly correlated with tumor location (hand vs. foot, OR: 0.25, *p* = 0.18) [72]. Another retrospective study of 25 patients treated at a single institution for NUM found no significant association between tumor location (hand vs. foot) and recurrence-free survival; 17/25 (68%) of NUM were located on the foot [73].

In a retrospective study of 244 patients with primary ALM, cases were classified by location as either hand-acral melanoma (H-AM) or foot-acral melanoma (F-AM) to determine whether prognosis, characterized by disease-free survival (DFS) in months, differed by tumor location. There was no statistically significant difference in prognosis for patients with H-AM vs. patients with F-AM (35 vs. 48, *p* = 0.1) [74]. Patients also showed a similar proportion of metastases for H-AM vs. F-AM (20.5% vs. 21%). [74]. This study did not specify how many NUM cases were included.

## 7. Pediatric NUM

**Figure 10:**
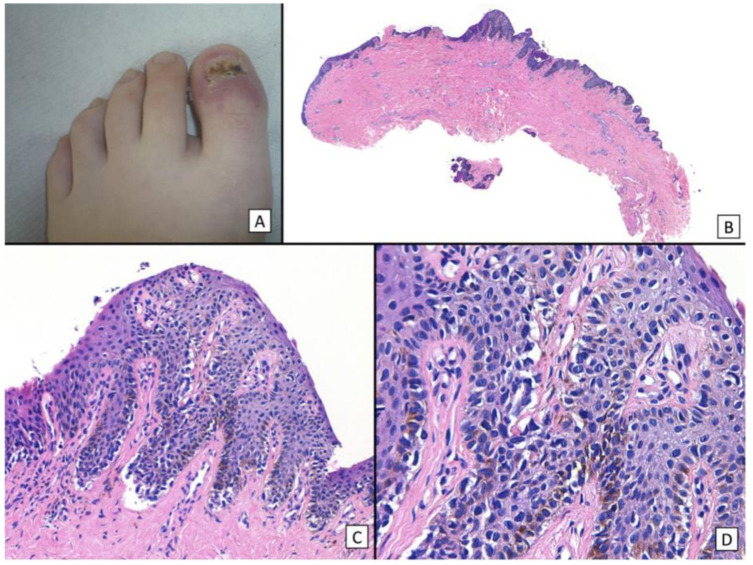
Subungual melanoma in situ (subungual lentiginous melanocytic proliferation with atypia). (**A**), A 13-year-old girl was referred to complete treatment of a lesion diagnosed as subungual melanoma in situ. The clinical examination revealed the nail bed to show no signs of persistence of the tumor after complete excision of the nail unit. (**B**), Panoramic view of a transverse biopsy of the matrix showing subepidermal fissures and intraepithelial cell proliferation. (**C**), Greater detail (×200) shows a lentiginous proliferation of atypical melanocytes, with the formation of fissures between the epithelium and underlying dermis and the focal suprabasal ascent of melanocytes, which completely replaces the keratinocytes in the basal layer. (**D**), High magnification (×400) reveals the atypical cellular characteristics of the proliferation: large melanocytes with pyknotic and pleomorphic nuclei and suprabasal ascent in some areas. Reproduced with permission from ref. [75].

**Figure 11:**
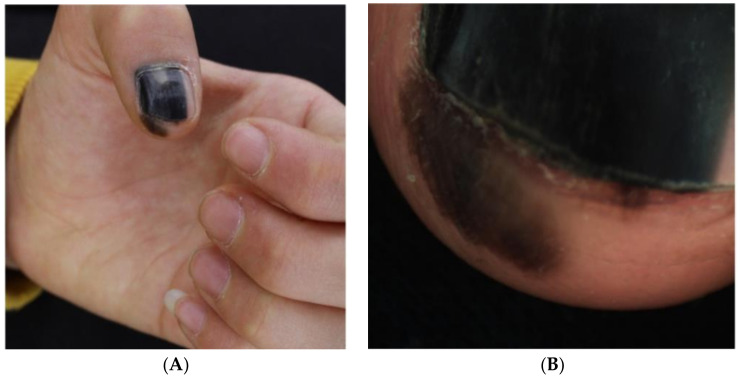
13-year-old girl presented with a painless black macule affecting her left thumbnail, later diagnosed as subungual melanoma. Clinical features of melanonychia in this case. (**A**), A homogenous black macule involving 70% of the nail plate, with a hypomelanotic lesion of proximal nail plate and irregular pigmentation extended to the hyponychium. (**B**), Dermoscopic features (polarized light) of the distal nail plate and hyponychium. Reproduced with permission from ref. [76].

**Figure 12:**
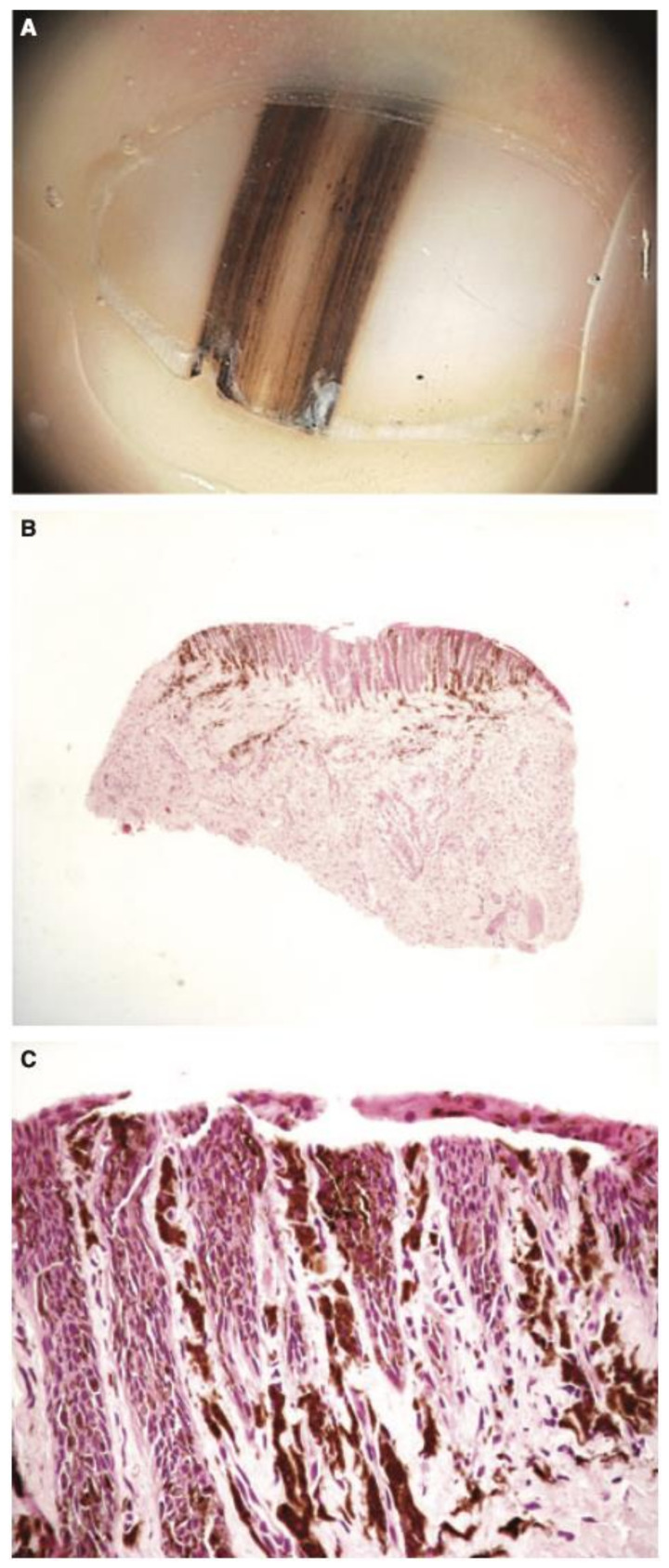
(**A**), 6-month-old boy with a band of longitudinal melanonychia of the first right toe. Dermatoscopy shows a dark-brown longitudinal band with lines exhibiting irregular coloration, spacing, and thickness. (**B**), Low-power view of the heavily pigmented matrix lesion. (**C**), High-power view of the mainly lentiginous proliferation of large, atypical melanocytes. Reproduced with permission from ref. [77].

**Figure 13:**
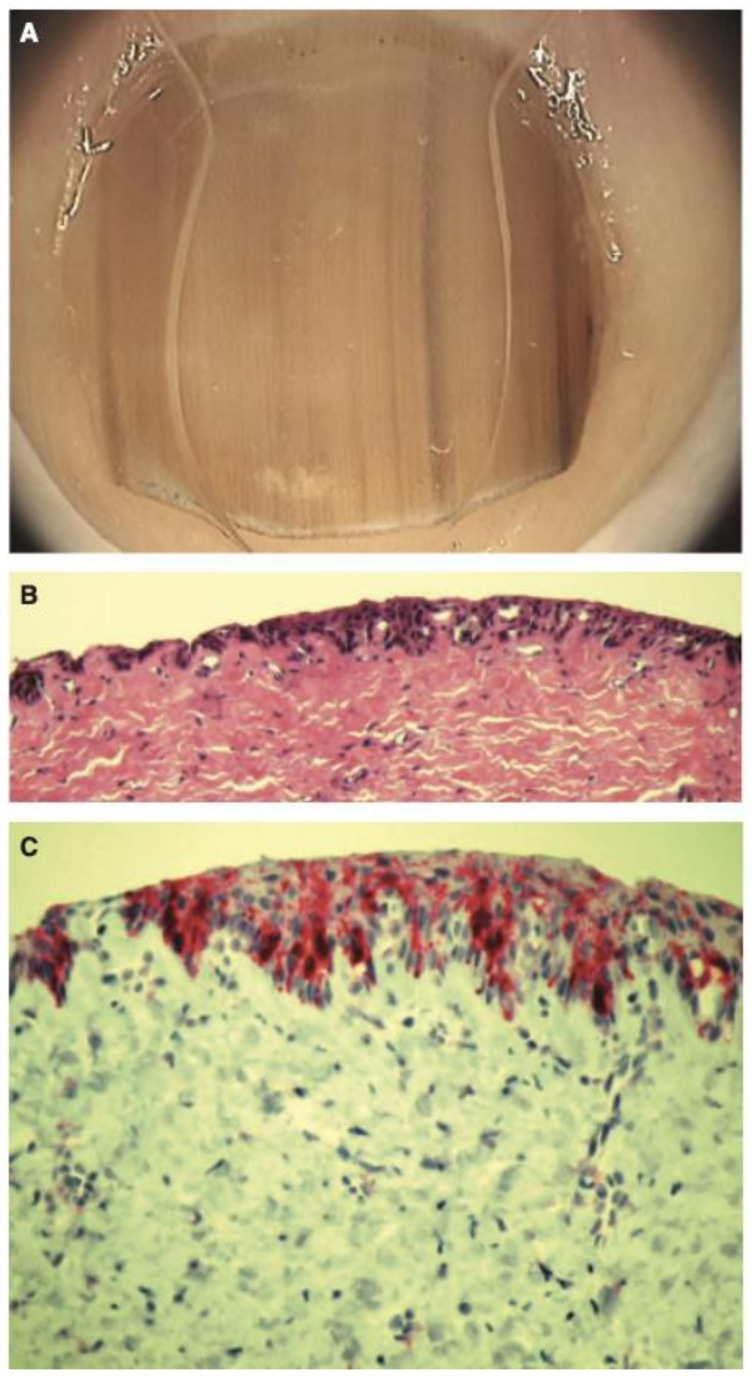
(**A**), 11-year-old girl with a longitudinal melanonychia of the second right fingernail. Dermatoscopy shows a pale brown background with lines of irregular coloration, spacing, and thickness. (**B**), Scanning view of large irregularly spaced melanocytes. (**C**), MelanA stain reveals abnormal positive melanocytes. Reproduced with permission from ref. [77].

Despite the rising rate of NUM diagnoses in adults, NUM diagnoses in pediatric patients are extremely rare [22]. Only 21 cases of NUM in children have been reported to date, with only 4 cases diagnosed as invasive and no instances of metastasis or death; there is controversy surrounding these cases, their pathology, and whether they are true cases of melanoma [75] (Figure 10, Figure 11, Figure 12 and Figure 13). All figures we include of pediatric cases were reported and described as NUM, although there may be disagreement about these diagnoses.

### 7.1. Pediatric NUM: Clinical Presentation & Dermoscopy

Diagnosis is challenging since no clear criteria have been established to differentiate between melanocytic activation, nail unit nevi, and NUM in children. While certain features (i.e., band width, pigment involving the nail folds) may guide the decision to perform a biopsy of LM to rule out NUM in adults, benign pediatric melanonychias may manifest similarly to adult NUM [75,78,79]. Pediatric nail unit nevi most often involve the thumbnail and may appear with a dark brown to black band, the band width being >3 mm, involve the entire nail plate with pigment extending to the proximal nail fold, and have irregular bands on dermoscopy [78]. This makes clinical and dermoscopic interpretation of LM in children challenging [31].

The most frequent cause of LM in childhood is a nail matrix nevus, in contrast to adult LM, which is most commonly caused by melanocytic activation [75,80,81,82]. In a retrospective study of 40 children less than 16 years who had biopsies, lentigo or nevus accounted for 77.5% of cases, with no patients diagnosed with NUM [83]. In a literature review of pigmented nail disorders affecting adults and children, nail unit nevi represent up to 12% of LM in adults compared to 50% in children, while nail unit lentigines represent 9% of LM in adults compared to 30% in children [51,83].

In a cohort study of 30 children 18 years and younger with LM who underwent nail matrix biopsy, histopathologic diagnoses included nail unit lentigo (n = 20), nail unit nevus (n = 5), and atypical melanocytic hyperplasia (n = 5); no cases were diagnosed as NUM [84]. Eight cases presented with pigmented bands with a width equal to or greater than 3 mm; seven cases presented with multiple colors or shades; and 8 cases showed Hutchinson or pseudo-Hutchinson sign; in 10 of the cases, the bands had evolved either in width or color by the time of clinical evaluation [84]. In a retrospective study comparing clinical and histopathologic features of 28 biopsy-proven nail matrix nevi in children (n = 20) and adults (n = 8), melanonychia was on average wider in children compared to adults (47% vs. 14% of nail, *p* = 0.028); Hutchinson sign was present in seven (35%) of the pediatric cases and none of the adult cases [85]. In these studies, many pediatric cases showed clinical features that would typically be alarming in adults, yet were benign diagnoses for all pediatric patients.

### 7.2. Diagnosis

Diagnosis and management of NUM in children remain controversial; differences in opinion on the exact diagnosis, lack of complete evidence in publications, and absence of aggressive clinical course in all cases contribute to ongoing discussions about the accuracy of diagnosing NUM in children [75,80]. One of the unknowns is at what age a person should be considered a child vs. an adult for purposes of assessing LM. The onset of puberty may potentially mark this delineation; however, this has not been studied. Another question is how LM is managed as a person progresses from childhood to adulthood, and whether the risk is higher or lower in these patients compared to new onset LM in an adult.

Diagnosis can pose a problem for dermatopathologists when making a diagnosis of nail unit melanoma in children, as well-established criteria are not present, and very few dermatopathologists have substantial experience with these challenging lesions.

### 7.3. Histopathology

Furthermore, histopathological features of NUM used as indicators in adults, such as prominent lentiginous growth, prominence of single melanocytes, or cytologic atypia, may be associated with benign diagnoses in children [76]. Other benign melanocytic cutaneous lesions in children, such as Spitz nevi, often have atypical architectural characteristics or cytologic atypia, but follow no aggressive clinical course [75]. This has led more researchers and pathologists to support the notion that atypical lentiginous melanocytic hyperplasia in children does not carry the same malignant potential as in adults [75].

In a cohort study examining 11 cases of nail unit atypical melanocytic proliferations presenting as LM in patients ages 2–19 with median width of 4 mm and a history of widening or darkening over time, 2 cases were diagnosed as in situ NUM, while the remaining 9 cases were diagnosed as atypical junctional melanocytic hyperplasia [86]. All cases demonstrated atypical histopathologic features overlapping with characteristics of adult NUM, such as poor circumscription, single-cell growth, and pagetoid scatter. Eight cases (73%) showed focal junctional nesting and three cases (27%) showed confluence. All cases showed nuclear atypia, either with a nuclear enlargement (73%), hyperchromasia (64%) and/or angulation (55%) [86]. This study examined four cases using fluorescence in situ hybridization (FISH); one of the in situ NUM cases had a 11q13 (CCND1) gain of function mutation, a finding that has been similarly reported in other cutaneous melanomas in children [86]. Since there is limited experience and data with FISH testing in pediatric nail melanocytic lesions, physicians should use caution when interpreting these results.

### 7.4. Management

There is no consensus on the management of LM in children, particularly because the predictive scoring models of NUM in adults have not been validated in children [87]. Nail matrix biopsy and histopathologic evaluation can be considered for pediatric LM cases that progress atypically with sudden changes such as rapid progression of an isolated band to total melanonychia; however, nail matrix nevi may present similarly [75,88].

Physicians may choose to use a different surgical approach in children compared to adults due to differences in how melanocytic lesions may expand in the nail unit. In adults, lesions are mostly confined to the matrix, and sampling from this area will usually give the correct diagnosis and does not cause persistence or recurrence. In children, lesions such as nevi may extend to other areas such as the proximal nail fold and hyponychium. A retrospective study analyzing the clinicopathologic features of nail matrix nevi from 20 children and 8 adults found that melanonychia was wider among children vs. adults (*p* = 0.002) [89]. Another retrospective study examining the dermoscopic features of nail matrix nevi from 56 children and 34 adults found that pseudo-Hutchinson sign was detected more in children (*p* = 0.004) and pigmentation tended to be broader in children, although not statistically significant [90]. Sampling the matrix only in children may consequently lead to insufficient tissue for diagnosis, with a greater risk for the persistence or recurrence of lesions. Depending on clinical presentation, some pediatric dermatologists may opt to send larger specimens for histopathologic review.

For less concerning LM, physicians should monitor with close follow-up. Conservative management follow-up every 6 months with clinical examination, dermoscopy, and photography is the standard of care; still, some dermatologists argue that the “wait and see” approach is not appropriate for all cases and may cause delayed diagnosis based on previous cases [20,78].

Although rare, it is important that physicians remain aware that NUM in the pediatric population is possible and that a nail matrix biopsy should be performed if there is a concern.

## 8. Conclusions

Detection, diagnosis, and treatment of NUM pose many challenges for physicians. Approximately two-thirds of NUMs present clinically as LM with a wide differential diagnosis. Diagnostic delay is common and may be secondary to suboptimal biopsy technique; misinterpretation of histopathology; and lack of physician or patient knowledge on clinical presentation. Dermoscopy can be extremely valuable for identifying common “alarm signs” in adults, although histopathological confirmation is needed in order to diagnose NUM. Treatment of NUM remains challenging because of the unique anatomy of the nail unit and its surrounding structures, and requires highly experienced physicians who have trained in this niche area; surgeons may have to account for bone invasion and make decisions about amputation at various joint levels. En bloc excision is favored over disarticulation for NUM in situ, with similar mortality and better quality of life for patients. Recent advances in treatment options include the use of targeted and immune systemic therapies, although further research is needed in order to better characterize NUM-specific response rates. Physicians should evaluate LM with a higher index of suspicion in adults compared to children. NUM in the pediatric population is extremely rare, and accurate diagnosis in previously reported cases is controversial. Our review highlights the recognition of warning signs for NUM that will hopefully translate into improved outcomes for patients with NUM.

## Figures and Tables

**Table 1:** Main approaches for treatment of NUM.

**Surgical**
Digit Amputation
En Bloc Excision
Mohs Micrographic Surgery
**Targeted Therapies**
Tyrosine Kinase Inhibitors
Dasatinib
Imatinib mesylate
Nilotinib
Sunitinib
High-dose interferon alfa-2b
BRAF Inhibitors
Vemurafenib
Dabrafenib
Encorafenib
MEK Inhibitors
Trametinib
Cobimetinib
**Immunotherapies**
PD-1 Inhibitors
Nivolumab
Pembrolizumab
Toripalimab
CTLA-4 Inhibitors
Ipilimumab
Tremelimumab

**Table 2:** Grouped efficacy and survival data for medical therapeutic modalities in patients with AM, ALM, and NUM * [65].

Therapy	#Studies	ORR, %	DCR, %	mOS, Months	mPFS, Months
CTLA-4 Inhibitors					
					
Ipilimumab	7	0–25.0	7.1–29.8	7.16–95.0	2.1–2.73
Tremelimumab	1	10.7	UK	12.6	UK
PD-1 Inhibitors					
					
Nivolumab	2	19.0	UK	14.03–25.8	6.56
Pembrolizumab	3	15.8–25.0	35.7–38.2	12.1	2.8
Toripalimab	2	14.0–23.1	46.2–57.5	16.9	3.2
Tyrosine Kinase Inhibitors					
					
Dasatinib	2	UK	UK	12.6–21.1	1.84–2.8
Imatinib mesylate	3	22.0	53.5	7.5–14.0	3.5
Nilotinib	5	16.7–26.2	47.6–77.8	4.3–18.0	2.5–4.2
Sunitinib	2	8.0	44.0	7.7	UK

AM: acral melanoma; ALM: acral lentiginous melanoma; NUM: nail unit melanoma; ORR: overall response rate; DCR: disease control rate; mOS: median overall survival; mPFS: median progression-free survival; UK: unknown. * All data has been adapted from a previous review and reported in ranges for multiple studies if information was available.

## Data Availability

No new data were created or analyzed in this study. Data sharing not applicable to this article.
